# Health Emergency and Disaster Risk Management: Five Years into Implementation of the Sendai Framework

**DOI:** 10.1007/s13753-020-00274-x

**Published:** 2020-04-30

**Authors:** Natalie Wright, Lucy Fagan, Jostacio M. Lapitan, Ryoma Kayano, Jonathan Abrahams, Qudsia Huda, Virginia Murray

**Affiliations:** 1 Global Public Health, Public Health England, London, SE1 8UG UK; 2 Disaster Risk Management and Resilience Unit, Health Security Preparedness Department, World Health Organization, Geneva, 1211 Switzerland; 3 WHO Centre for Health Development (WHO Kobe Centre), Kobe, Hyogo 651-0073 Japan

**Keywords:** Data collection, Disaster risk management, Health emergencies, Research networks, Sendai Framework

## Abstract

The Sendai Framework for Disaster Risk Reduction 2015–2030 recognizes health at the heart of disaster risk management (DRM) at the global policy level. Five years on, it has catalyzed the rapid development of the field of Health Emergency and Disaster Risk Management (Health EDRM) by providing a mandate for building partnerships as well as enhancing scientific research. Key milestones achieved include publication of the World Health Organization’s Health EDRM Framework, development of the WHO Thematic Platform for Health EDRM and the WHO Health EDRM Research Network, and further application of health information principles to DRM. Furthermore, health actors at all levels have continued to engage in the Sendai Framework processes and have had a key role in its implementation and proposed monitoring. There have been significant gains made through the partnership of health and DRM, but the relationship has not been without its challenges. Many national, regional, and global initiatives continue to operate with a lack of consistency and of linkages to respond to the Sendai Framework’s call for embedding health resilience in DRM, and conversely, embedding DRM in health resilience. Overcoming this hurdle is important, and doing so will be a key marker of success of the next 10 years of partnership under the Sendai Framework.

## Introduction

Five years have passed since the adoption of the Sendai Framework for Disaster Risk Reduction 2015–2030 (UNDRR 2015) by 187 United Nations (UN) member states at the Third UN World Conference on Disaster Risk Reduction in Japan in March 2015 (UNDRR 2015).^1^ With the synchronous adoption of the Sendai Framework as a landmark UN agreement that links closely with the Sustainable Development Goals (SDGs) (UNGA 2015), Paris Climate Agreement (UNFCCC 2015), and the outcomes of the World Humanitarian Summit (UNGA 2016a) and Habitat III (UNGA 2016b) the aim has been to develop dynamic, local, preventive, and adaptive governance systems at the global, national, and local levels. These landmark UN agreements aim to develop and lead a global process that can create a rare but important opportunity to build coherence across different but overlapping policy areas (Murray et al. 2017). Taken together, these frameworks aim for a more complete agenda for action that spans health, development, humanitarian action, disaster risk management (DRM), and climate change adaptation.

Communicating and understanding the value of the Sendai Framework across all sectors, including for and to health professionals, is critical for progress on the health priorities. The Sendai Framework recognizes that by reducing and managing conditions of hazard, exposure, and vulnerability—while building the capacity of communities and countries for prevention, preparedness, response, and recovery—losses and impacts on health from disasters can be more effectively alleviated through a multisectoral approach rather than focusing exclusively on emergency response.

While there were several references to health in the Hyogo Framework for Action 2005–2015 (UNDRR 2005), the adoption of the Sendai Framework marks the first time that the fields of health and DRM have been substantially interwoven at the global multisectoral policy level. This article builds on previous efforts to discuss this merger (Aitsi-Selmi and Murray 2015; Aitsi-Selmi et al. 2015b; Maini et al. 2017) and explores its effects 5 years on, not just in terms of global policy and partnership, but also in terms of the effects for scientific research and data in the field of DRM.

## Leading into the Sendai Framework

The health sector has a long-standing history of excellence in developing evidence-based policy and practice, with globally recognized organizations dedicated to this endeavor.

Cochrane is a leading example of such an organization. It is a non-profit institution, set up in 1992, which works with over 130 contributors globally to produce high quality, accessible health information, free from commercial interests. It advocates the principles of evidence-based medicine as critical to health policy and practice, and in assessing the effectiveness of interventions for disease prevention, treatment, and rehabilitation (Turner et al. 2017). Cochrane Reviews are recognized as the highest standard of systematic reviews in health care and are published online in the free-access Cochrane Database of Systematic Reviews in the Cochrane Library (Cochrane 2020).

Evidence Aid is another example of a leader in the field of evidence-based practice in the health sector. The organization, which has charitable status in the United Kingdom, uses evidence from systematic reviews to provide up-to-date advice on interventions in the context of planning for or responding to disasters, humanitarian crises, and other major healthcare emergencies (Khalid et al. 2019). It seeks to identify which interventions are most and least effective—including those that may unintentionally cause harm.

The UN system and its member states have achieved globally agreed guidelines for DRM since 1990 (UNDRR 2019). Historically, the health impacts of disasters were poorly reflected in this international dialogue. The Yokohama Strategy and Plan of Action for a Safer World (International Decade for Natural Disaster Reduction 1994) did not mention health or health care facilities at all. Its successor, the Hyogo Framework, mentions health as a sector and health care facilities three times, but not as an explicit goal or outcome of DRM.

The World Health Organization (WHO) and its partners have a set of programs for managing and preventing health emergencies that predate the adoption of the Sendai Framework. The WHO Thematic Platform for Health EDRM (Health Emergency and Disaster Risk Management) was launched by WHO and UNDRR on the International Day for Disaster Reduction, 14 October 2009. The impetus for its launch came from both the 2008–2009 World Disaster Reduction Campaign on Hospitals Safe from Disasters (UNGA 2008) and the 2009 Global Platform for Disaster Risk Reduction (UNGA 2009) when participants supported a proposal to establish a thematic platform dedicated to protecting public health from the risks and consequences of emergencies and disasters and in support of the Hyogo Framework.

There are several other key programs. In particular, the International Health Regulations (IHR) (2005) constitute an important legal tool for UN member states, which is designed to facilitate the prevention of and the response to acute public health risks that have the potential to cross borders and pose global threats to health (WHO 2016a; for wider perspectives on international law, see Aronsson-Storrier 2020). Also, the Inter-Agency Standing Committee (IASC) Emergency Response Preparedness (ERP) draft for field testing sets standards for risk analysis and monitoring, and minimum and advanced preparedness including contingency planning, with the aim of optimizing the speed and volume of critical assistance provided immediately following the onset of a humanitarian emergency (IASC 2015).

Building on all this work, the Sendai Framework puts health risks and health resilience at the heart of global DRM efforts. It advocates for involving health sectors throughout planning for emergency proactive and reactive measures globally, as well as highlighting the critical role of science and technology. There are 38 references to health, including links to epidemics and pandemics, alongside several references to the 2005 IHR (WHO 2016a) and to rehabilitation as part of disaster recovery. Some examples are:

To enhance the resilience of national health systems, including by integrating disaster risk management into primary, secondary and tertiary health care, especially at the local level; developing the capacity of health workers in understanding disaster risk […] and supporting and training community health groups in disaster risk reduction approaches in health programmes, in collaboration with other sectors, as well as in the implementation of the International Health Regulations (2005) of the World Health Organization. (Paragraph 30 i)

People with life-threatening and chronic disease, due to their particular needs, should be included in the design of policies and plans to manage their risks before, during and after disasters, including having access to life-saving services. (Paragraph 30 k)

To promote the resilience of new and existing critical infrastructure, including water, transportation and telecommunications infrastructure, educational facilities, hospitals and other health facilities, to ensure that they remain safe, effective and operational during and after disasters […]. (paragraph 33 c)

Supporting the convergence of global health and DRM agendas, examples of organizations long involved in DRM that are now supporting the Sendai Framework are:

- The World Association for Disaster and Emergency Medicine (WADEM) seeks to improve the delivery of prehospital and emergency care worldwide during disasters and other emergencies. It is the oldest international emergency and disaster medicine organization and has members from 55 countries and multiple disciplines, such as medicine (including veterinary medicine), nursing, psychology, sociology, emergency management and academia from both governmental and nongovernmental organizations (WADEM 2020). At its 2015 Congress, the WADEM adopted the Cape Town Statement, which explicitly supports the Sendai Framework and its implementation in the health emergency and disaster risk management domain. As a result, the WADEM was one of the organizations that were most rapid in adopting and supporting the implementation of the Sendai Framework and its impacts on health EDRM (Emergency and Disaster Risk Management) evidence, policy, and practice engagement at its various meetings and congresses since 2015.
- The International Association of National Public Health Institutes (IANPHI), started in 2006, links, supports, and strengthens the institutes responsible for public health in countries worldwide, particularly in low resource settings. Its focus areas include outbreak investigation and control, disease surveillance, health promotion, and emergency response (Pekka and Jeffrey 2017). The IANPHI network agreed to form a new DRM Group at their 2016 Annual Meeting in Shanghai, October 17–21, to raise new awareness of global policy and action toward DRM and possible engagement opportunities for national public health institutes. Since then, work has been ongoing to embed DRM approaches into the work of national public health institutes.
- The International Federation of Environmental Health (IFEH) is an umbrella organization whose members are the national environmental health organizations of countries, as well as universities and associated members. The IFEH represents an estimated 50,000+ professionals and academics from 43 countries working in environmental health, mainly at local, regional, and state governmental levels (IFEH 2019). In May 2017, the IFEH endorsed the principles and agenda behind the Sendai Framework.
- The International Council of Nurses (ICN) is a global federation of over 130 national nursing associations that endeavors to ensure quality nursing care universally, sound health policies, the development of nursing knowledge, competencies, and skills, and the worldwide presence of a competent, professional, fulfilled, and respected nursing workforce (ICN 2020). The ICN sees the nursing profession as an essential partner in the response and prevention of disasters and considers that it remains an underused resource in the field of DRM. In 2019, the ICN updated their Nurses and Disaster Risk Reduction, Response and Recovery policy statement in line with the Sendai Framework, highlighting that “nurses must be involved in the development and implementation of disaster risk reduction, response and recovery policies at the international level” (ICN 2019, p. 2).

Being part of the UN system, the World Health Organization contributed to the lead-into the Sendai Framework and is now an active supporter and implementer of it.

## World Health Organization

Since the adoption of the Sendai Framework, the WHO has increasingly sought to strengthen its approach to health emergencies and DRM. This includes embedding all-hazard emergency risk management at the heart of its General Programme of Work, as well as the development of several key initiatives designed to support the generation and application of research, knowledge, and expertise to the global prevention and management of outbreaks and other health emergencies.

### General Programme of Work

The WHO reports in its 13th General Programme of Work (GPW 13) for 2019–2023 that major global health gains have been made in recent years, yet complex, interconnected threats, such as poverty and inequality to conflict, and poor governance remain. The GPW 13, which sets out WHO’s strategic direction for the next 5 years and was approved by the Seventy-First World Health Assembly in resolution WHA71.1 on 25 May 2018, is informed by the UN’s 2030 Agenda for Sustainable Development, and in particular SDG 3: to ensure healthy lives and promote wellbeing for all at all ages (WHO 2018).

The focus of GPW 13 is to deliver meaningful, large-scale, country-level impact to promote health, improve health security, and serve vulnerable communities. A key pillar of this vision is to reduce the risks and impacts of all types of health emergencies. The GPW 13 recognizes that “the world faces threats from high-impact health emergencies (epidemics, pandemics, conflicts, natural and technological disasters) and the emergence of antimicrobial resistance” (WHO 2018, p. 1). It has at its heart a set of interconnected strategic priorities and goals through the “triple billion” target, which includes: 1 billion more people benefitting from universal health coverage; 1 billion more people better protected from health emergencies; and 1 billion more people enjoying better health and well-being (WHO 2018).

In the context of health emergencies, the WHO will “work with Member States and partners to increase all-hazards health emergency detection and risk management capacities across all phases of risk prevention and detection, emergency preparedness, response and recovery through the implementation of the IHR (2005) and the Sendai Framework for Disaster Risk Reduction” (WHO 2018, p. 23). The GPW 13 goes on to state that “WHO’s approach to health emergencies is described in the results framework of the health emergencies programme. It seeks to ensure that:

- Populations affected by health emergencies have access to essential life-saving health services and public health interventions;
- All countries are equipped to mitigate risk from high-threat infectious hazards;
- All countries assess and address critical gaps in preparedness for health emergencies, including in core capacities under the 2005 IHR and in capacities for all-hazard health emergency risk management;
- National health emergency programmes are supported by a well-resourced and efficient WHO Health Emergencies Programme.” (WHO 2018, p. 24)

### Thematic Platform for Health Emergency and Disaster Risk Management

Recognizing the complexity of delivering on the ambitions for health in the Sendai Framework, the WHO, together with national ministries of health, UN agencies, and partners, has sought to build greater coherence and interlinkages among these actors and initiatives through the WHO Thematic Platform for Health EDRM, which has the intent to promote health resilience in a consistent manner, both within and beyond the health sector. The Thematic Platform recognizes that engagement and collaboration with the wider health system and other sectors (especially at local levels) is critical in the prevention of health risks, as many of the necessary actions to reduce hazards and vulnerabilities rests with the activities of other sectors. The Thematic Platform was also actively engaged during the negotiations of the Sendai Framework, providing advice on health to member states, and playing a crucial advocacy role.

Since 2015, the Thematic Platform has provided advice and recommendations on health issues to member states on the implementation of the Sendai Framework and advanced efforts to mainstream DRM within the work of the WHO and other health partners, as well as promoted health within DRM. Some of the key outputs of the Thematic Platform have been facilitating inputs from more than 50 experts to develop and revise a series of fact sheets on various aspects of health emergency and disaster risk management, organize key workshops and forums at the Global Platform for Disaster Risk Reduction, and provide health-related inputs to UNDRR reports and thematic conferences (for example, science and technology).

The Thematic Platform is guided by, and supports the implementation of, the Sendai Framework, the SDGs, and the Paris Agreement, along with the IHR, WHO resolutions, and other regional and global frameworks. Some initiatives, such as the collaboration on disaster education for medical studies, have grown extensively and include links with the International Federation of Medical Students Associations and many academic partners including the Centre for Research Disaster and Emergency Medicine at the University of Eastern Piedmont in Italy.

### Other Programs for Health Emergencies

The WHO, along with its partners, has a number of other ongoing programs that it implements for health emergencies that support the implementation of the IHR, Sendai Framework, Pandemic Influenza Preparedness Framework, and other global policies. These include:

- The WHO *Strategic Framework for Emergency Preparedness*, which lays out the principles and constituents of effective country health emergency preparedness (WHO 2017);
- The WHO’s *An R&D Blueprint for Action to Prevent Epidemics*, a global strategy and preparedness plan that supports the rapid implementation of research and development activities during epidemics (WHO 2016b); and
- The IHR (2005)* Monitoring and Evaluation Framework* that includes annual reporting by states parties, simulation exercises, after action reviews, and voluntary joint external evaluation, which is a collaborative process to assess a country’s capacity to adhere to the IHR requirements (WHO 2016c).

Other global initiatives include the Global Health Security Agenda (GHSA), a non-binding coalition of countries and organizations working to strengthen capacity to respond to infectious disease threats and promote health security as a national and global priority (Bali and Taaffe 2017).

In their own rights, these initiatives have taken (and continue to take) important steps towards improving the prevention, detection and response to health emergencies globally. Furthermore, they point to the fact that the sector is implementing many aspects of the Sendai Framework already, given its references to biological hazards and the IHR (2005). There remains a lack of joined up working to fully respond to the Sendai Framework’s call for promoting health resilience in a consistent manner both within the health sector itself and more broadly across sectors. The 2019 WHO Health Emergency and Disaster Risk Management Framework aims to facilitate this consistency of approach.

### Health Emergency and Disaster Risk Management Framework

Drawing on the learning from 5 years of implementing the health aspects of the Sendai Framework by ministries of health and partners, as well as successive global and regional frameworks on emergency preparedness, DRM, and the IHR of 2005, the WHO launched a new framework for Health EDRM at the Global Platform for Disaster Risk Reduction in 2019 (WHO 2019). This framework addresses the many issues raised by the Sendai Framework and is designed to provide an overarching frame to bring together the many vital initiatives to deliver disaster risk management and increase preparedness for public health emergencies. The Framework also aims to embed a Health EDRM within existing health systems, thus enabling a greater emphasis on risk prevention and building health resilience at community and national levels, community resilience, along with preparedness, response, and recovery.

The Health EDRM Framework has a clear vision—the “highest possible standard of health and well-being for all people who are at risk of emergencies, and stronger community and country resilience, health security, universal health coverage and sustainable development” (WHO 2019, p. x) It aims to strengthen capacity, within and beyond the health sector, to tackle the health impacts of all types of emergencies and disasters, as well as to work to reduce the health risks of future events. The framework is derived from the disciplines of risk management, emergency management, epidemic preparedness and response, and health systems strengthening. It places an emphasis on multisectoral action and, importantly, recognizes the importance of building resilience as part of the wider health system strengthening approach and the journey to achieve universal health coverage in all country contexts (see Box 1). It is fully consistent with existing DRM and health emergency policies and seeks to provide a framework for aligning these in future.

**Box 1:** Functions and Components of the World Health Organization’s Health Emergency and Disaster Risk Management Framework

•*Policies, strategies and legislation* defines the structures, roles and responsibilities of governments and other actors for Health EDRM; includes strategies for strengthening Health EDRM capacities.	
•*Planning and coordination* emphasizes effective coordination mechanisms for planning and operations for Health EDRM.	
•*Human resources* includes planning for staffing, education and training across the spectrum of Health EDRM capacities at all levels, and the occupational health and safety of personnel.	
•*Financial resources* supports implementation of Health EDRM activities, capacity development and contingency funding for emergency response and recovery.	
•*Information and knowledge management* includes risk assessment, surveillance, early warning, information management, technical guidance and research.	
•*Risk communications* recognizes that communicating effectively is critical for health and other sectors, government authorities, the media, and the general public.	
•*Health infrastructure and logistics* focuses on safe, sustainable, secure and prepared health facilities, critical infrastructure (e.g. water, power), and logistics and supply systems to support Health EDRM.	
•*Health and related services* recognizes the wide range of health-care services and related measures for Health EDRM.	
•*Community capacities for Health EDRM* focuses on strengthening local health workforce capacities and inclusive community-centred planning and action.	
•*Monitoring and evaluation* includes processes to monitor progress towards meeting Health EDRM objectives, including monitoring risks and capacities and evaluating the implementation of strategies, related programmes and activities.	

*Source* WHO (2019, p. x–xi).

At the time of writing, the Health EDRM Framework was recently published, and is in the earliest stages of implementation. Plans are in place for wide engagement with ministries of health for training and development on strategies and programs through WHO regional and country offices and partners. Specific actions to support operationalization of the Health EDRM Framework include accelerating implementation of the National Action Planning for Health Security (NAPHS) process, strengthening an all-hazards approach to strategic emergency risk assessments and emergency response planning, mainstreaming DRM in all health policies and programs, and supporting improved Sendai Framework reporting by ministries of health. Going forward, it will be vital to continue to proactively implement the Framework, particularly at the country level, and to evaluate its impact globally.

### Regional Approaches

There are a number of regional approaches established by WHO Regional Offices, which have referenced the Sendai Framework in efforts to bring together health and DRM. For example, in 2015, the Regional Committee for South East Asia approved the Resolution on Response to Emergencies and Disaster, which reflects emergency and disaster risk management as a flagship priority area (WHO 2015). It makes explicit reference to the Sendai Framework as well as the IHR and SDGs, and reaffirms the need for DRM policies across sectors and at all levels of government, to ensure the effective response to disasters and other emergencies.

This response was taken one step further by the WHO Regional Office for the Americas/Pan-American Health Organization (PAHO). Responding to the ambitious agenda set out in the Sendai Framework as well as reforms to the outbreak response capacity of the WHO, PAHO launched the Regional Plan of Action for Disaster Risk Reduction 2016–2021 (PAHO 2016). Approved by the member states in September 2016, the plan provides an operational framework to guide the implementation of DRM policies and programs in the health sector. It contains four strategic lines of action that are consistent with the Sendai Framework and are aimed at reducing the health impacts of disasters and emergencies: (1) recognizing disaster risks; (2) strengthening governance of disaster risk management; (3) promoting safe and smart hospitals; and (4) strengthening the sector’s capacity for emergency and disaster preparedness, response, and recovery.

A progress report, published in 2018, showed that at least 23 member states of PAHO had undertaken, or were in the process of undertaking, a national evaluation of disaster risk in the health sector. Furthermore, there had been an increase in the number of countries with full-time staff and an allocated budget for health DRM (PAHO 2018). This represents an important step forward in health emergency and disaster risk management, translating global ambitions on health and DRM into a concrete and contextualized set of actions for implementation. However, more action on the multisectoral dimensions of Health EDRM is needed. The progress report also found only eight countries had a multisectoral plan for recovery after emergencies and disasters—just over 25% of those that responded. Moving ahead, it is critical to align health and DRM strategies at regional, national, and local levels, ensuring that health is represented in disaster risk reduction plans (Target (e)) and national adaptation plans.

## The Science and Technology Agenda

Embedding health into DRM at the policy level, with its principles of evidence-based policy making, as shown in Sects. 2 and 3, has had the resultant effect of bringing in new scientific methods and rigor.

### The Sendai Framework’s Science and Technology Agenda

Health based scientific research and outcomes are needed to identify needs and knowledge gaps. Working in partnership with the UNDRR Science and Technical Advisory Group (UNDRR STAG) and linking health to DRM to implement the Sendai Framework will have significant impact particularly when the call for action in Paragraph 25(g) of the Sendai Framework (UNDRR 2015) to “Enhance the scientific and technical work on disaster risk reduction” is followed. In order to achieve this, health, science, and technology communities and networks should mobilize and strengthen existing capacities and initiatives to working to co-design, co-produce, and co-deliver new knowledge that is readily available and accessible.

Coherence between the Sendai Framework, the SDGs, the Paris Agreement, the New Urban Agenda, and the World Humanitarian Summit, and the role of science in their implementation support the implementation of the post-2015 framework from the local to the global scale. The 2015 UNDRR STAG report recommends the delivery of outputs as detailed in Box 2.

**Box 2:** Recommended Outputs in 2015 from the United Nations International Strategy for Disaster Reduction, Science and Technical Advisory Group

•*Assessment* of the current state of data, scientific knowledge and technical availability on disaster risks and resilience (what is known, what is needed, what are the uncertainties, etc.);	
•*Synthesis* of scientific evidence in a timely, accessible and policy-relevant manner;	
•*Scientific advice* to decision-makers through close collaboration and dialogue to identify knowledge needs including at national and local levels, and review policy options based on scientific evidence; and	
•*Monitoring and review* to ensure that new and up-to-date scientific information is used in data collection and monitoring progress towards disaster risk reduction and resilience building.	
In addition, two cross-cutting capabilities need to be strengthened:	
•*Communication and engagement* among policy-makers, stakeholders in all sectors and in the science and technology domains themselves to ensure useful knowledge is identified and needs are met, and scientists are better equipped to provide evidence and advice; and	
•*Capacity development* to ensure that all countries can produce, have access to and effectively use scientific information.	

*Source* Aitsi-Selmi et al. (2015a).

The use of scientifically derived evidence by government is not without its difficulties, however, particularly in the context of today’s geopolitical climate and ongoing debate about the role of scientific experts in policy making (Ross et al. 2016; Pearce et al. 2018). Engaging policymakers in science does not just mean making research results available. It also means helping them understand the implications and working with them to decide how to respond, and what further research or other activity is needed.

Many of these challenges were discussed at the 2016 UNDRR Science and Technology Conference on the Implementation of the Sendai Framework for Disaster Risk Reduction 2015–2030 (Aitsi-Selmi et al. 2016; Dickinson et al. 2016) as well as at the meeting in Tokyo in 2017 (Science Council of Japan 2017). Other international science meetings have been held across the world with specialist health groups engaging to consider how best to support the need for evidence to inform policy and practice. Possible strategies for achieving this include building on existing programs that are known to be effective, strengthening relationships across sectors nationally and globally, and considering reworking policy narratives (Bardosh et al. 2017).

### WHO’s Science and Technology Agenda

As part of the solutions identified, representatives of the health community and WHO worked together to set up a research network for Health EDRM. The WHO Health EDRM Research Network links to the WHO Thematic Platform for Health EDRM and was initially proposed in 2015 and formally launched in 2018.

The Research Network aims to serve as an international multistakeholder and interdisciplinary platform to exchange information, share views, and advise WHO in the area of Health EDRM research and evidence-related activities in general and on the following issues in particular:

- The development of information sharing platforms on research and activities relating to Health EDRM being undertaken across the world;
- The development of partnerships between stakeholders to enhance the scientific and technical work on Health EDRM, influence the international Health EDRM research agenda, and advocate for greater health input within the wider disaster risk reduction community;
- The provision of technical advice and review of guidance for Health EDRM research initiatives and the standardisation of related programmes; and
- The provision of support to the WHO in other aspects of its Health EDRM work, where applicable.

Also promoted by the Research Network are partnerships among stakeholders to enhance the scientific and technical work on Health EDRM, influence the international research agenda, and advocate for greater health input within the wider DRM community. The Research Network comprises the core group, participants, and a wider information-sharing network. WHO headquarters and Regional Offices are involved in the operation of both the Thematic Platform and the Research Network, and work together to discuss research needs, facilitate international research collaboration, and improve knowledge management. The WHO Kobe Centre acts as the secretariat for this platform.

The unique value of the Research Network lies in its ability to leverage capacity across the six WHO regions, enabling (1) strengthening of the evidence base on Health EDRM on a global scale; and (2) the rapid dissemination of emerging evidence to member states. The ability for rapid and widespread dissemination facilitates the implementation of Health EDRM policy and practice founded on the most robust and up-to-date evidence available. This, in turn, should maximize the opportunities at national, regional, and community levels to implement the most effective evidence-based programs known that reduce the risks and impacts of disasters to the world’s most vulnerable populations. In doing so, this should enable countries to strengthen their capacities in Health EDRM and ultimately contribute to one billion more people being safe during emergencies.

The Research Network and other key partners have identified, through a meeting convened by the WHO Kobe Centre in 2018, five research priority areas before, during, and after emergencies and disasters:

- *Health data management*
  This research priority focuses on developing tools and methodologies to support timely and accurate data collection, analysis, and dissemination in recognition of the critical importance of reliable data in the provision of effective health support in disaster relief and recovery.
- *Mental health and psychosocial support*
  Emergencies and disasters can place significant and persistent mental health pressures on those affected, including the responders. Understanding the most effective approaches to embedding mental health and psychosocial support into disaster response is an essential part of Health EDRM.
- *Addressing the health risks and needs of subpopulations, including health literacy*
  Understanding the health vulnerabilities, capacities, and inequities of communities and specific subpopulations is critical to disaster risk management.
- *Health workforce development for Health Emergency and Disaster Risk Management*
  This is an overarching research theme of Health EDRM that encompasses all thematic research areas in acknowledging that effective disaster response and risk management hinges on highly skilled, trained, and supported workforces (including community actors).
- *Research methods and ethics*
  WHO Kobe Centre coordinates the development of the WHO Guidance on Research Methods for Health EDRM—comprehensive guidance (in textbook form) for designing, conducting, and reporting research, including research ethics, in emergencies and disasters (Aung et al. 2019).

A detailed description of the expert meeting and its findings was published in a series of papers (Aung et al. 2019; Généreux et al. 2019; Kayano et al. 2019; Kubo et al. 2019). In 2019, the WHO Kobe Centre launched the first call for proposals on Health EDRM addressing the first four priority areas. The development of the WHO Guidance on Research Methods for Health EDRM, which addresses the fifth research priority, is currently underway.

The WHO Guidance on Research Methods for Health EDRM is presented as a resource textbook comprising 42 chapters authored by 92 contributors from 17 countries around the world, with a wide variety of Health EDRM disciplines. The book is structured around six sections: introduction; identifying and understanding the problem; assessing the problems and developing a scoping study; study design; special topics to demonstrate research processes and benefits; and how to become a researcher. It includes an extensive series of case studies, 26 from individual countries and 33 from more than two countries or of global relevance. The book is edited by six leading experts in Health EDRM and has over 60 peer reviewers from 21 countries. It is expected to be published in 2020 on open access via the WHO Kobe Centre website.

The publication of this guidance initiative is particularly useful for all health professionals in Health EDRM, given the overall lack of research in the field of Health EDRM and the consequently limited evidence base. It will facilitate, enable, and enhance Health EDRM researchers to increase the depth and breadth of evidence available, which will inform and foster evidence-based policy and practice in the field. It will also enable the harmonization of Health EDRM research with universal terms; the use of mechanisms to facilitate and speed up the ethical review process; increased community participation and stakeholder involvement in generating research ideas and in assessing impact evaluation; and the development of reference materials such as consensus statements.

## Data, Monitoring, and Reporting

The adoption of the Sendai Framework by the UN member states includes agreement on seven global targets to assess progress in DRM:

- Substantially reduce global disaster mortality by 2030, aiming to lower the average per 100,000 global mortality rate in the decade 2020–2030 compared to the period 2005–2015;
- Substantially reduce the number of affected people globally by 2030, aiming to lower the average global figure per 100,000 in the decade 2020–2030 compared to the period 2005–2015;
- Reduce direct disaster economic loss in relation to global gross domestic product (GDP) by 2030;
- Substantially reduce disaster damage to critical infrastructure and disruption of basic services, among them health and educational facilities, including through developing their resilience by 2030;
- Substantially increase the number of countries with national and local disaster risk reduction strategies by 2020;
- Substantially enhance international cooperation to developing countries through adequate and sustainable support to complement their national actions for implementation of the present Framework by 2030;
- Substantially increase the availability of and access to multi-hazard early warning systems and disaster risk information and assessments to people by 2030. (UNDRR 2015, p. 12)

UNDRR has developed technical guidance for monitoring and reporting on national progress in achieving these targets and has prepared a web-based tool to support this reporting (UNDRR 2017) as well as a training package for member states. Most member states have a Sendai Framework Monitoring National Focal Point with responsibility for national reporting on the Sendai Framework targets. Many of these targets (and many of the associated 38 indicators) have clear links to the health impacts of disasters. For example, there are health specific indicators on mortality (A-2), people injured and ill (B-2), damage and destruction of health facilities (D-2), and disruption to basic health services (D-7). Access to these health data is integral to timely, accurate, and complete reporting to the Sendai Framework Monitor.

Because ministries of health hold relevant data related to health outcomes, risks, and capacities, it is vital that ministries of health are engaged with the Sendai Framework Monitoring National Focal Point and work collaboratively with relevant partners to ensure comprehensive and accurate reporting. To date, this engagement across member states is mixed, with 48% (93/195) reporting against mortality targets in 2017 and 45% (87/195) in 2018 (UNDRR n.d.). Going forward, it is vital to continue working to maximize engagement across all member states. The WHO Technical Guidance Notes on Sendai Framework reporting for ministries of health is one tool that aims to facilitate this engagement, and provides guidance for the health sector on its role in data collection and reporting, as well as detailed descriptions of health specific indicators (WHO due for publication in early 2020).

**Fig. 1:**
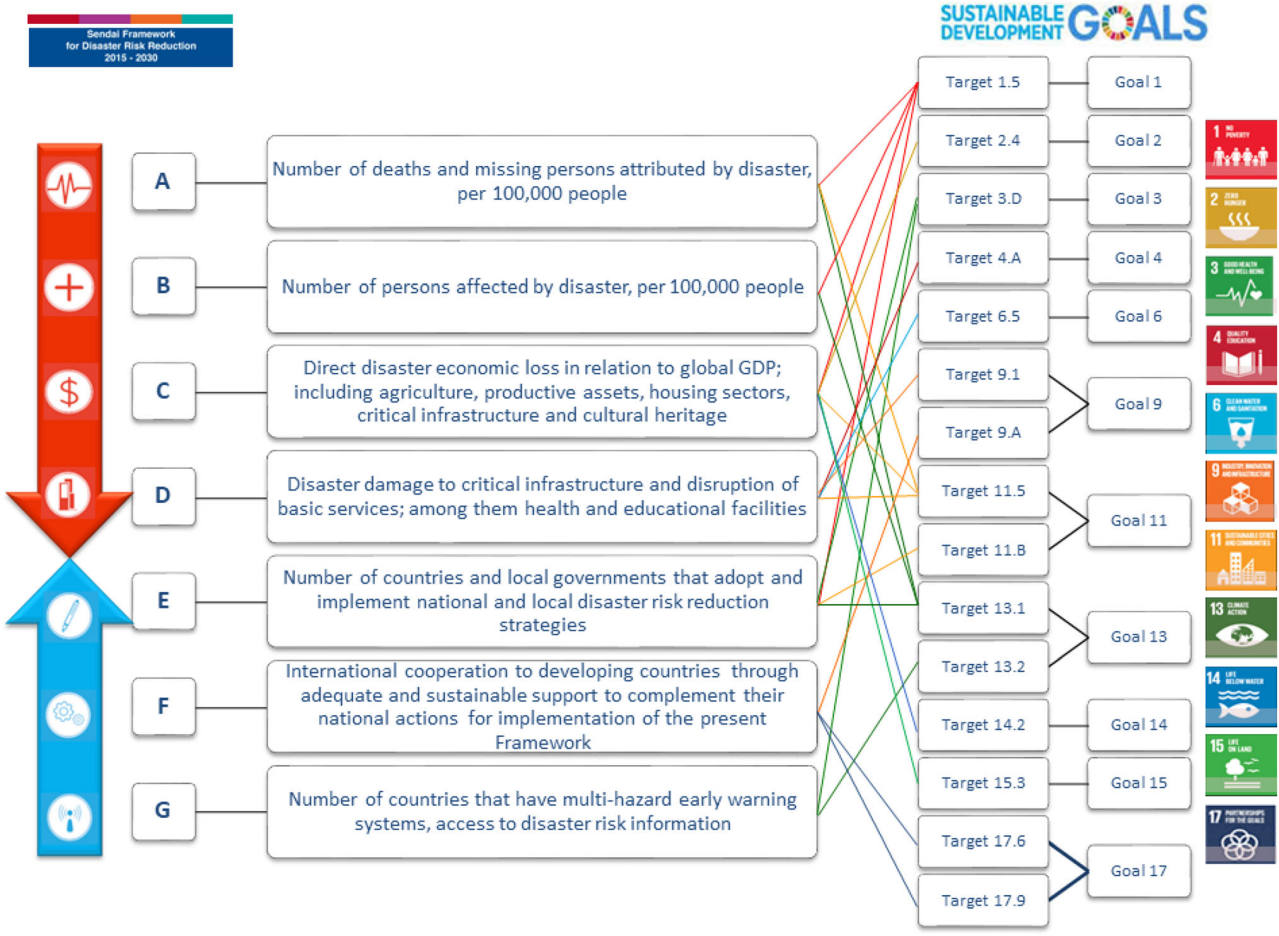
Links between sendai framework targets and sustainable development goals

Health sector reporting to the Sendai Framework Monitor will enable ministries of health to measure annual trends of the effects of emergencies and disasters on health, review progress in strengthening capacities, and prioritize areas for further action. There are also clear links to reporting on the SDGs. Figure 1 highlights the web of links between targets of the Sendai Framework and the SDGs, including the SDG target 3d: Strengthen the capacity of all countries, in particular developing countries, for early warning, risk reduction, and management of national and global health risks.

There are several benefits to building coherence between reporting for the Sendai Framework Monitor and the SDGs. These include increasing awareness at national and subnational levels of government on how the two frameworks align; facilitating key partnerships, which help avoid duplication of data collection and reporting; promoting accountability; and enabling the development of overarching strategies for collective action on findings in order to maximize gains for both the health and disaster risk management sectors.

There are several developments that could support the engagement of ministries of health in supplying data for inclusion in the member state reporting process. Member states should consider organizing health sector training—regionally, nationally, and subnationally—in methods to improve monitoring and reporting on Sendai targets. They should also consider further review of UNDRR technical guidance and training for the development of disaster data bases. In the medium and longer terms, ministries of health could consider strengthening national and subnational capacities for civil registration and vital statistics, and developing national case registries for mortality and morbidity related to hazardous events, including emergencies and disasters (Green et al. 2019).

## Conclusion and Way Forward

The growing recognition of health as a core dimension in DRM has catalyzed the development of Health EDRM, a field that encompasses emergency and disaster medicine, DRM, humanitarian action, global health security, adaptation to climate change, and resilience of health systems, communities, and countries. This has led to developing the WHO Health EDRM Framework, which aligns with the WHO’s GPW 13: a critical tool for WHO member states to set and approve the priorities of the organization, define the targets to be delivered, and to monitor their achievements. The GPW 13 is structured around three strategic priorities, one of which specifically addresses health emergencies and aims to build and sustain the resilient health systems required to reduce the risks of epidemics and other health emergencies.

The WHO Health EDRM Research Network was established in direct response to contributing to the Sendai Framework in general and the Sendai Framework Monitor in particular. The network aims to strengthen research in Health EDRM and to promote the sharing of knowledge and evidence globally. This marks a crucial step towards enhancing evidence-based policy making and practice, and is a key enabler for delivering truly joined-up efforts.

Despite the perceived success of the Framework and the Research Network, many national, regional, and global initiatives continue to operate with a lack of consistency and harmony in their response to the Sendai Framework’s call for the embedding of health resilience in DRM, and conversely, the embedding of DRM in health resilience (Wisner 2020). The health sector is implementing many aspects of the Sendai Framework already, but there is a weakness in the way it recognizes, records, and reports this implementation. The policies, programs, and actions taken by governments, WHO, and partners that are aimed at reducing the risks and impacts of emergencies and disasters show the range of health sector actions that could be considered as evidence of implementing the Sendai Framework.

Going forward, effective and efficient coordination of work is critical, building on emergency preparedness and response, while simultaneously putting a greater emphasis on prevention and recovery. This requires close collaboration with the wider health system and engagement with other sectors—a key public health approach—given that many of the actions to prevent health risks (by reducing hazards and vulnerabilities) rest with the activities of other sectors. Ministries of health and their partners are encouraged to engage further with the application of the Health EDRM Framework, with the WHO Thematic Platform for Health EDRM and its associated Research Network, and in reporting to their National Focal Points for the Sendai Framework Monitor. This will enable ministries of health to measure the effects of emergencies and disasters on health, review progress in strengthening capacities, and prioritize areas for further action. The engagement of the ministries of health will therefore be a key marker of success in the effort to nurture the partnership of health and DRM under the Sendai Framework.

## Footnotes

1 The United Nations Office for Disaster Risk Reduction (UNDRR) was previously known as the United Nations International Strategy for Disaster Reduction (UNISDR). The term UNDRR is used throughout this article irrespective of the term used to describe the agency at the time referred to.
